# In Vitro Preservation of Transgenic Tomato (*Solanum lycopersicum* L.) Plants Overexpressing the Stress-Related SlAREB1 Transcription Factor

**DOI:** 10.3390/ijms18071477

**Published:** 2017-07-21

**Authors:** Ayed M. Al-Abdallat, Rida A. Shibli, Muhanad W. Akash, Manar Rabbaa, Tamara Al-Qudah

**Affiliations:** 1 Department of Horticulture and Crop Science, Faculty of Agriculture, the University of Jordan, 11942 Amman, Jordan; r.shibli@ju.edu.jo (R.A.S.); makash@ju.edu.jo (M.W.A.); rabbaco@yahoo.com (M.R.); 2 Hamdi Mango Center for Scientific Research, the University of Jordan, 11942 Amman, Jordan; T.alqudah@ju.edu.jo

**Keywords:** abscisic acid, encapsulation-dehydration, in vitro preservation, *Solanum lycopersicum*, transcription factor, V-cryoplates

## Abstract

In vitro preservation of transgenic tomato lines overexpressing the stress-responsive transcription factor *SlAREB1* was studied by using slow growth and cryopreservation techniques. Slow growth preservation was performed by using different concentrations of sucrose (0, 100, 200, 300 mm) and abscisic acid (0, 4, 8, 12 μm) in Murashige and Skoog (MS) media, while cryopreservation was conducted by using encapsulation dehydration, V-cryoplates and seeds. Significant differences were observed between tested lines grown on MS media supplemented with 200 mm sucrose where transgenic lines overexpressing *SlAREB1* showed improved growth when compared with negative control. The addition of abscisic acid (ABA) to the preservation media affected negatively transgenic lines growth and development when compared with ABA-free media. In encapsulation dehydration, non-cryopreserved transgenic lines overexpressing *SlAREB1* pretreated in 0.8 M sucrose for 1 day and subjected to different dehydration periods showed significantly higher survival percentages when compared with negative control. For V-cryoplates technique, cryopreserved transgenic lines overexpressing *SlAREB1* treated in 0.3 M sucrose for 3 days with or without cold acclimatization showed significantly higher survival percentages when compared with the negative control. Seed cryopreservation was performed successfully with a clear reduction in germination percentage in transgenic lines overexpressing high levels of *SlAREB1*. In conclusion, transgenic tomato lines overexpressing *SlAREB1* were found to improve tolerance against different abiotic stresses associated with different in vitro preservation protocols.

## 1. Introduction

Tomato (*Solanum lycopersicum* L.) is a warm season plant, which is considered sensitive to cold and under severe frost conditions the entire plant could be destroyed. Such sensitivity to cold and other related abiotic stresses can be overcome by several physiological and biochemical processes such as the accumulation of abscisic acid (ABA), osmolytes and late embryogenesis abundant proteins and growth retardation [1]. Abscisic acid is known to regulate several physiological processes including seed germination, stomata closure and tolerance to different abiotic stresses [2]. ABA has been reported to enhance tolerance to different abiotic stresses including low temperature, drought and salinity stresses in several plant species.

Understanding the genetic basis of stress tolerance mechanisms in plants may offer the opportunity to develop new varieties with improved adaption to different stresses including cold and drought. In this perspective, an important step in controlling plant tolerance responses is the transcriptional activation or repression of stress-responsive genes [3]. Transcriptional regulation of stress tolerance genes is largely mediated by the specific recognition of *cis*-acting promoter elements by trans-acting sequence specific DNA-binding proteins known as transcription factors. In addition, such responses are mediated mainly through ABA-dependent and ABA-independent signals that regulate the expression of stress responsive genes involved in conferring stress tolerance phenotypes [3]. The basic strategy of generating tolerant plants is based on the activation of either one or both pathways through the introduction of key regulatory genes that are directly involved in activating molecular mechanisms controlling abiotic stress tolerance [4]. The ABA responsive element binding (AREB) factors (also known as the ABF for ABA binding factors) are members of the bZIP family of transcription factors that specifically bind to *ARE cis*-elements found in the promoter region of many ABA-responsive genes [5]. The overexpression of the *AREB* factors in different plant species improves freezing and dehydration tolerance [4]. In tomato, the overexpression of *SlAREB1* was found to increase drought and salt stress tolerance [6] as well as the induction of changes in organic acid accumulation and the expression of gene encoding enzymes involved in their synthesis [7].

The loss of valuable plant genetic resources has prompted international actions to preserve such valuable exotic plant material [8]. In vitro, preservation of plant germplasm can be divided into medium-term preservation (slow growth preservation) and long-term preservation (cryopreservation) [9]. In medium-term preservation, the plant material is subjected to growth retardation conditions that slow down plant growth and development in vitro. This includes growth under low temperatures, reduced light conditions and modification of gaseous environments, and the use of growth retardants such as ABA and osmoticums [10]. Medium-term preservation was used successfully for many plant species where regeneration potential could be restored [11]. Cryopreservation refers to the conservation of plant germplasm for indefinite periods by keeping it in ultra-low temperature conditions such as liquid nitrogen (−196 °C) [12]. Under such conditions, the biochemical processes, metabolic activities, and cell division of the cryopreserved biological material will be arrested [13]. The cryopreserved material can retain its biological activities as soon as it is allowed to re-grow under suitable conditions. Cryopreservation in liquid nitrogen is the only currently available method that ensures safe, cost-effective and long-term conservation of vegetatively propagated plants and for plants with recalcitrant seeds [10,14]. Cryopreservation is performed by using different methods such as encapsulation dehydration, vitrification, encapsulation-vitrification and cryoplates [15]. For instance, the cryopreservation of *Solanum phureja* shoot tips was achieved successfully by using the encapsulation dehydration approach [16]. In 2014 and 2015, Coste et al. [17,18] succeeded in the cryopreservation of five tomato cultivars by using encapsulation dehydration and droplet vitrification. They found that the preculturing of shoot tips in sucrose plays an important role in increasing the efficiency of cryopreservation by enhancing the resistance to desiccation and liquid nitrogen exposure. Recently, a new cryopreservation technique was developed by using aluminium cryoplates that could be either based on vitrification (V-cryoplates) or dehydration-(d-cryoplates) and both were found to give high survival and regrowth percentages after cryopreservation when compared to other methods [19,20,21]. The utilization of seeds as a plant material in cryopreservation was used successfully in different plant species including tomato [22,23].

Previously, the cryopreservation of transgenic plants was performed successfully with no major alternations in cryopreserved material phenotype or genotype after their regeneration [24,25,26]. For instance, transgenic chrysanthemum plant with improved trehalose accumulation had higher regeneration percentages when compared with wild type [26]. In this study, in vitro preservation of transgenic lines overexpressing the stress-related *SlAREB1* transcription factors was studied by using slow-growth and cryopreservation approaches. For slow-growth preservation, the effect of different levels of sucrose and ABA on microshoot development after 12 weeks of culture was studied. Cryopreservation of transgenic lines was studied by using encapsulation-dehydration, V-cryoplates and seeds. To the best of our knowledge, no previous reports describing the effect of stress-related transcription factors on in vitro preservation of the tomato plant exist.

## 2. Results

### 2.1. Overexpression of SlAREB1 in Tomato

To test whether *SlAREB1* can improve in vitro preservation of tomato plants, an overexpression construct with *SlAREB1* CDS (GenBank accession number: AY530758) under the control of *35S CaMV* promoter was introduced into tomato cv. Money maker. Only 5 independent transgenic lines were produced and were confirmed to be positive based on hygromycin selection. Three of them were found to carry a single insertion event as revealed by real time PCR analysis (data not shown). The transgenic lines grew normally when compared with tomato plants transformed with empty plasmid and this is consistent with Orellana et al. (2010) [6], who showed that the majority of transgenic lines overexpressing *SlAREB1* had normal morphological phenotypes. Gene expression analysis using quantitative RT-PCR analysis in transgenic tomato plants overexpressing *SlAREB1* showed high expression levels of the targeted gene when compared with negative control plants (Figure S1). However, a clear difference in *SlAREB1* expression levels between the transgenic lines was obvious with SlAREB1#3 line showing the highest value. This particular line showed a delayed germination phenotype when compared with other tested lines. The T3 generations of two homozygous lines overexpressing different levels of *SlAREB1* (SlAREB1#2 and SlAREB1#3) and a single transgenic line harboring empty T-DNA were selected and used further for in vitro preservation experiments.

### 2.2. Slow Growth Preservation

**Figure 1:**
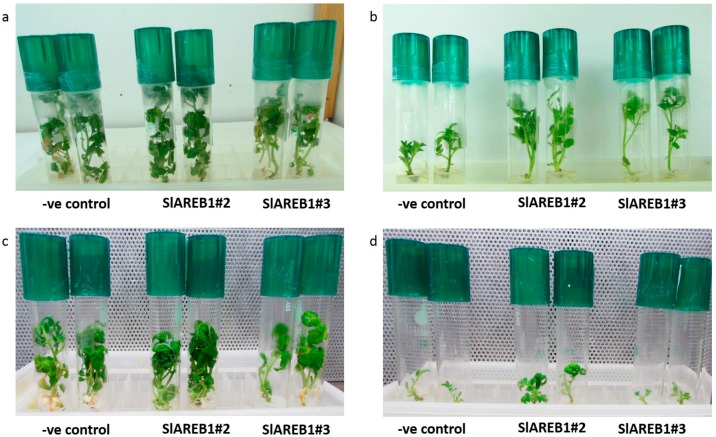
Slow growth preservation of two transgenic lines overexpressing *SlAREB1* (SlAREB1#2 and SlAREB1#3) and negative control after 12 weeks of culturing. (**a**) Growth on MS media supplemented with 100 mm sucrose without ABA; (**b**) Growth on MS media supplemented with 200 mm sucrose without ABA; (**c**) Growth on MS media supplemented with 100 mm sucrose and 4 µm ABA; (**d**) Growth on MS media supplemented with 100 mm sucrose and 8 µm ABA.

To test the effect of sucrose concentration on slow growth preservation of transgenic tomato lines, microshoots were incubated for 12 weeks on MS media supplemented with different levels of sucrose (0, 100, 200, 300 mm). Microshoots placed on media lacking sucrose showed a growth arrest phenotype where no significant changes in plant height, roots number and leaves number mean values were observed (Table 1). At 100 mm sucrose level, all tested plants showed normal growth and development with respect to plant height, leaves number and roots number, although no significant differences were observed between transgenic lines and negative control (Figure 1a; Table 1). Increasing sucrose concentration in the storage media to 200 mm significantly reduced growth in negative control microshoots when compared with transgenic lines overexpressing *SlAREB1* after 6 and 12 weeks of storage (Figure 1b; Table 1). The effect of 200 mm sucrose level on rooting was also obvious where a full inhibition of root formation was observed in negative control microshoots when compared with SlAREB1 transgenic lines. At 300 mm sucrose level, microshoots of transgenic line SlAREB1#3 significantly produced the highest mean values for plant height, root number and leaf number indicating its ability to grow under high osmotic stress conditions (Table 1). At the same level, the microshoots of transgenic line SlAREB1#2 showed growth retardation phenotype. For regrowth potential, the SlAREB1#3 microshoots were able to give the highest percentage after 12 weeks of culturing at 200 and 300 mm sucrose levels when compared with negative control (Table 2).

To test the effect of ABA concentration on slow growth preservation of transgenic tomato lines, microshoots were incubated for 12 weeks on MS media supplemented with different levels of ABA (0, 4, 8 and 12 µm). SlAREB1#3 microshoots had significantly lower roots and leaves number at 4 µm ABA level after 6 and 12 weeks of storage when compared with ABA-free media (Table 3). Furthermore, only SlAREB1#3 microshoots cultivated on MS media supplemented with 4 µm ABA level for 12 weeks showed a significantly lower plant height when compared with negative control and SlAREB1#2 microshoots. A decrease in microshoots height was recorded after 6 weeks of culturing when ABA was added to the media compared to the control (ABA-free media), which was more pronounced after 12 weeks only at high ABA levels (8 and 12 µm) (Table 3). For roots and leaves numbers, significant reductions in all tested lines were observed at high ABA levels (8 and 12 µm) after 6 and 12 weeks of storage when compared with ABA-free media. For regrowth potential, the addition of ABA to the preservation media didn’t affect the regrowth percentages of all tested lines when compared with ABA-free media (Table 2).

### 2.3. Cryopreservation

**Figure 2:**
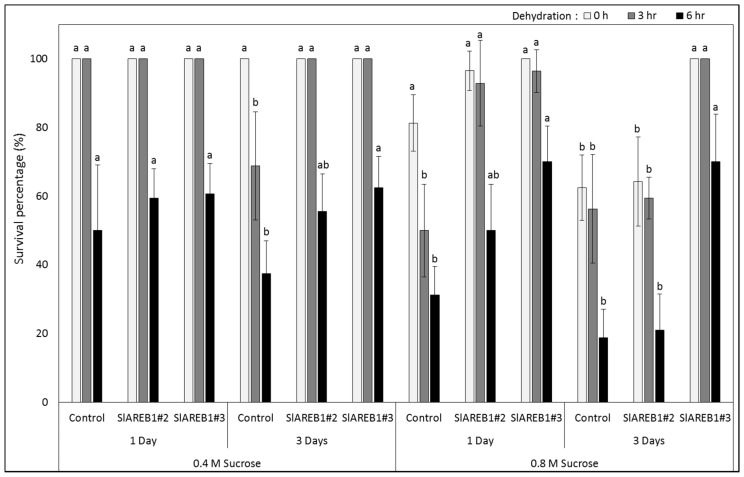
Survival percentages of encapsulated-dehydrated shoot tips of non-cryopreserved shoots tips (−LN) of negative control, SlAREB1#2 and SlAREB1#3 transgenic tomato plants as affected by air dehydration duration after pretreatment with 0.4 and 0.8 M sucrose concentration for one or three days (values are the mean ± SD). Based on Tukey’s HSD test, different letter indicates significant difference among transgenic lines (*p* < 0.05) for each combination of sucrose concentration, number of days, and air dehydration duration.

**Figure 3:**
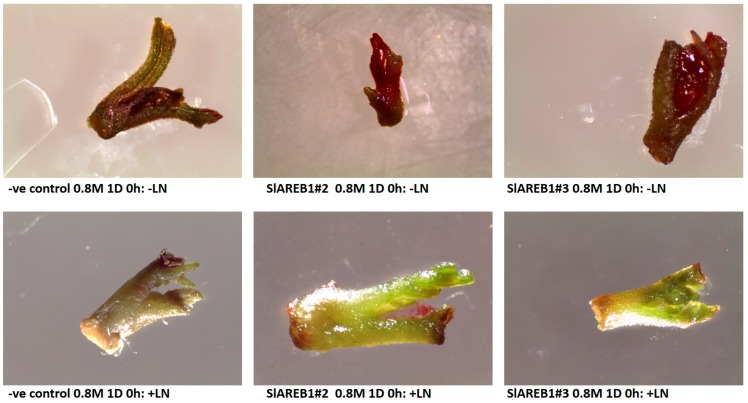
Survival of two transgenic lines overexpressing *SlAREB1* (SlAREB1#2 and SlAREB1#3) and negative control (-ve control) shoot tips after encapsulation dehydration treatment of 1 day (1 D) incubation in 0.8 M sucrose and 0 h dehydration with (+LN) or without liquid nitrogen (−LN).

The effect of cryopreservation on transgenic tomato lines was studied by using encapsulation-dehydration, V-cryoplates and seeds. For encapsulation-dehydration, microshoots of transgenic lines were subjected to different treatments including combinations of two sucrose levels (0.4 and 0.8 M), incubation period (1 and 3 days) and dehydration period (1, 3, 6 h) before subjecting them to liquid nitrogen or without (room temperature). Initially, the moisture content of treated beads was determined and found to decrease with increasing the period of dehydration (Figure S2). For encapsulated shoot tips without liquid nitrogen, a complete survival percentage (100%) was observed with negative control, SlAREB1#2, and SlAREB1#3 treated in 0.4 M sucrose for one day and dehydrated for 0 or 3 h and in 0.4 M sucrose for three days and dehydrated for 0 h (Figure 2). On the other hand, encapsulated shoot tips of negative control treated in 0.4 M sucrose for 3 days and dehydrated for 3 and 6 h produced lower survival percentages when compared with SlAREB1#2 and SlAREB1#3 shoot tips. Encapsulated shoot tips of negative control line treated in 0.8 M sucrose for 1 day with 0 h dehydration showed a slight reduction in survival percentages when compared with SlAREB1 transgenic lines but a clear reduction was observed in shoot tips dehydrated for 3 and 6 h (Figure 2 and Figure 3). On the other hand, non-cryopreserved encapsulated shoot tips of SlAREB1#3 transgenic line treated in 0.8 M sucrose for 3 days showed a significant increase in survival percentages when compared with negative control and SlAREB1#2 shoot tips.

**Figure 4:**
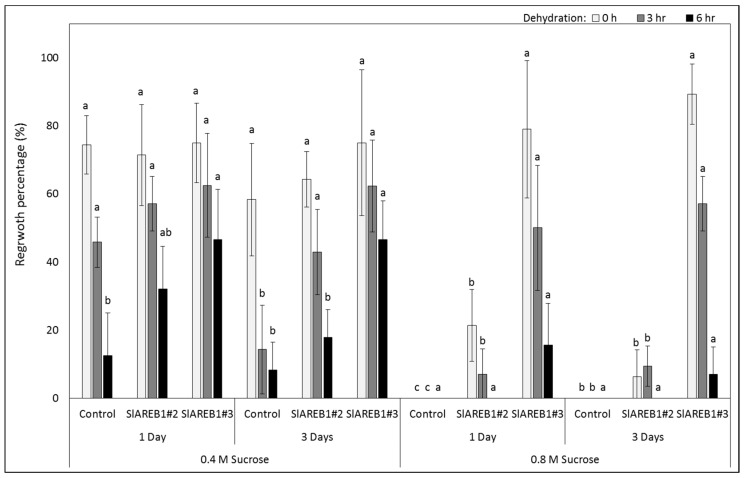
Regrowth percentages of encapsulated-dehydrated shoot tips of non-cryopreserved shoots tips (−LN) of negative control, SlAREB1#2 and SlAREB1#3 transgenic tomato plants as affected by air dehydration duration after pretreatment with 0.4 and 0.8 M sucrose concentration for one or three days (values are the mean ± SD). Based on Tukey′s HSD test, different letter indicates significant difference among transgenic lines (*p* < 0.05) for each combination of sucrose concentration, number of days, and air dehydration duration.

**Figure 5:**
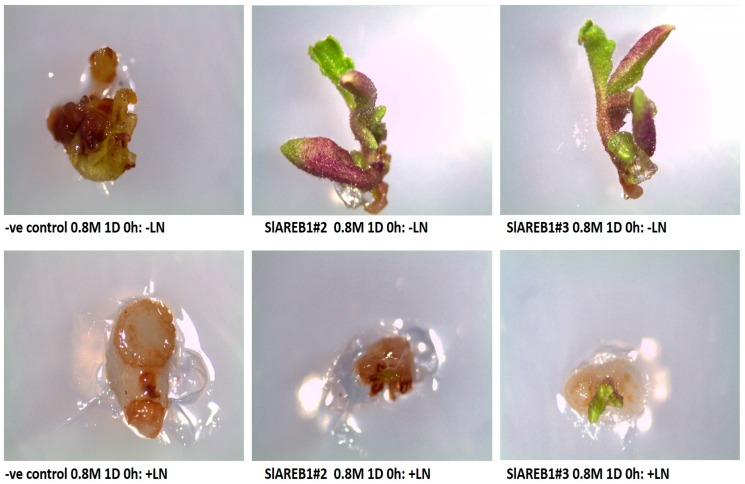
Regrowth of two transgenic lines overexpressing *SlAREB1* (SlAREB1#2 and SlAREB1#3) and negative control (-ve control) shoot tips after encapsulation dehydration treatment of 1 day (1 D) incubation in 0.8 M sucrose and 0 h dehydration with (+LN) or without liquid nitrogen (−LN).

In general, the highest regrowth percentages in the non-cryopreserved transgenic lines were observed in shoot tips treated in 0.4 M sucrose for one day and dehydrated for 0 h (Figure 4). The dehydration of shoot tips for 6 h resulted in significant reduction in regrowth percentages of all tested lines at 0.4 M sucrose level incubated either for 1 or 3 days when compared with 0 h. However, transgenic SlAREB1#2 and SlAREB1#3 shoot tips treated in 0.4 M sucrose for 3 days and dehydrated for 3 h showed significantly higher regrowth percentages when compared with negative control (Figure 4). Irrespective of incubation time and dehydration period, the treatment in 0.8 M sucrose resulted in the absence of regrowth in encapsulated-dehydrated negative control shoot tips (Figure 4 and Figure 5). On the other hand, transgenic SlAREB1#2 and SlAREB1#3 shoot tips treated in 0.8 M sucrose for 1 day and dehydrated for 0 h were able to regrow (Figure 4 and Figure 5). Meanwhile, SlAREB1#3 shoot tips treated in 0.8 M sucrose for 3 days and dehydrated for 0 and 3 h showed a significant ability to regrow when compared with other tested lines. On the contrary to non-cryopreserved treatments, neither survival nor regrowth were observed in all transgenic lines subjected to cryopreservation in liquid nitrogen (Figure 3 and Figure 5).

**Figure 6:**
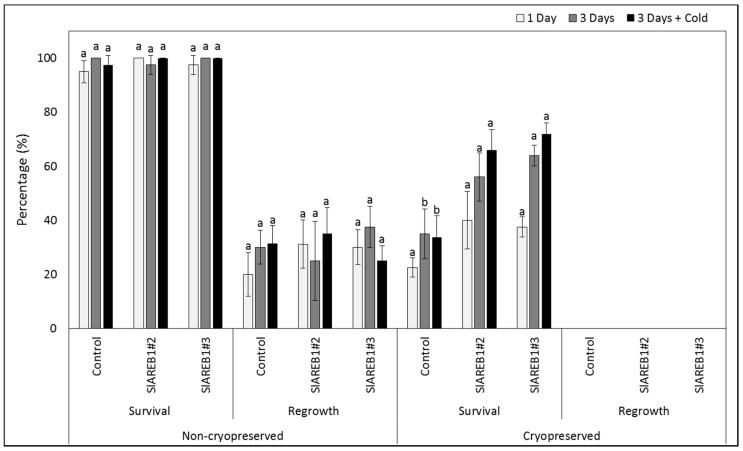
Survival and regrowth percentages of V-cryoplates treated non-cryopreserved and cryopreserved shoots tips of negative control, SlAREB1#2, SlAREB1#3 transgenic tomato plants as affected by pretreatment with 0.3 M sucrose concentration for one day or three days with or without cold acclimatization (values are the mean ± SD). Based on Tukey′s HSD test, different letter indicates significant difference among transgenic lines (*p* < 0.05) for each combination of cryopreservation and cold acclimatization treatment.

**Figure 7:**
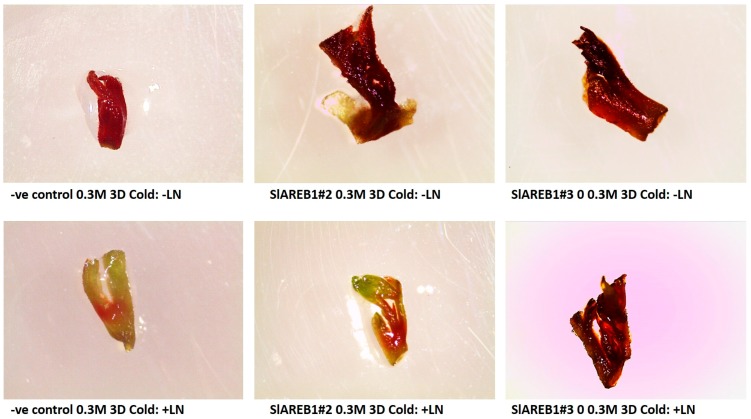
Survival of two transgenic lines overexpressing *SlAREB1* (SlAREB1#2 and SlAREB1#3) and negative control (-ve control) after V-cryoplates treatment of 3 day incubation in 0.3 M sucrose and cold acclimatization for 4 days with (+LN) or without liquid nitrogen (−LN).

**Figure 8:**
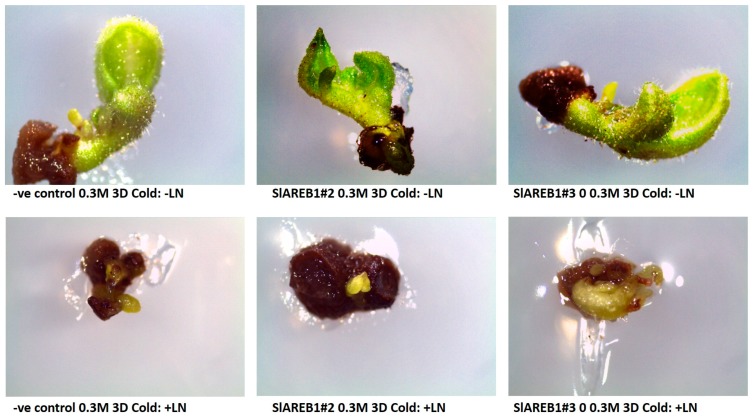
Regrowth of two transgenic lines overexpressing *SlAREB1* (SlAREB1#2 and SlAREB1#3) and negative control (-ve control) after V-cryoplates treatment of 3 day incubation in 0.3 M sucrose and cold acclimatization for 4 days with (+LN) or without liquid nitrogen (−LN).

To test the effect of cryopreservation using V-cryoplates technique on the transgenic tomato lines, shoot tips were treated in 0.3 M sucrose for two incubation periods (1 or 3 days) without cold acclimatization and in 0.3 M sucrose for three days with a cold acclimatization pretreatment for 3 days. The treated shoot tips were subjected then to either liquid nitrogen (cryopreservation) or kept at room temperature (without liquid nitrogen). Irrespective to incubation time or cold acclimatization, the V-cryoplates technique produced high survival percentages in all tested transgenic lines that were not subjected to liquid nitrogen treatment (Figure 6 and Figure 7). In addition, no major differences in regrowth percentages were observed between the treated non-cryopreserved transgenic lines (Figure 6 and Figure 8). Nevertheless, regrowth percentages of non-cryopreserved shoot tips were considerably lower than observed survival percentages (Figure 6). Cryopreserved shoot tips (with liquid nitrogen) of all transgenic lines showed lower survival percentages when compared with non-cryopreserved shoot tips (Figure 6 and Figure 7). On the other hand, cryopreserved transgenic SlAREB1#2 and SlAREB1#3 shoot tips treated in 0.3 M sucrose for 3 days with or without cold acclimatization showed significantly higher survival percentages when compared with the negative control. No regrowth of cryopreserved shoot tips was observed in all transgenic lines (Figure 6 and Figure 8).

**Figure 9:**
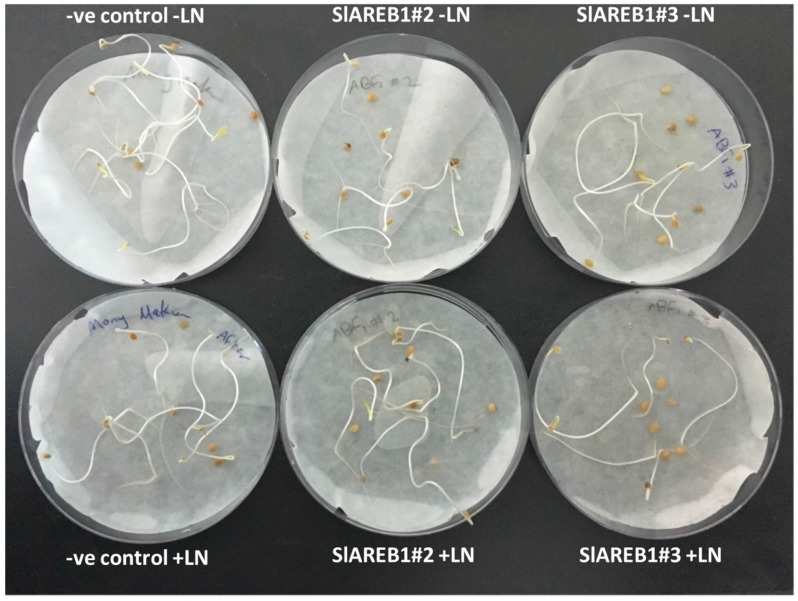
Seed germination of two transgenic lines overexpressing *SlAREB1* (SlAREB1#2 and SlAREB1#3) and negative control (-ve control) after 10 days of desiccation with silica gel and treatment with liquid nitrogen (+LN) or without liquid nitrogen (−LN).

For seed cryopreservation experiments, 100 seeds of each transgenic tomato line were subjected to dissection by using silica gel until they reached an equilibrium state where the initial fresh weight is equal to dry weight. Thereafter, the treated seeds were subjected then to either liquid nitrogen (cryopreservation) or kept at room temperature (without liquid nitrogen). Seed cryopreservation was performed successfully with the ability of all transgenic lines to germinate after liquid nitrogen treatment (Figure 9; Table 4). However, a significant difference exists between tested transgenic lines where SlAREB1#3 seeds produced lower percentages when compared with negative control and SlAREB1#2. This was also observed in non-cryopreserved seeds indicating that the reduced germination in SlAREB1#3 is most likely related to the genotype itself. This was further confirmed by performing a germination test on tested transgenic lines seeds (without silica gel treatment) incubated on filter paper with distilled water (data not shown).

## 3. Discussion

In vitro preservation of plants by using slow growth and cryopreservation techniques is considered a valuable tool for the conservation of genetic material outside their natural habitats. In this study, the effect of the tomato stress-responsive *SlAREB1* overexpression on slow growth and cryopreservation was studied. Previously, *SlAREB1* was found to improve drought and salt stress tolerance in tomato under normal growth conditions [6]. This was associated with the upregulation of several stress-responsive genes such as late embryogenesis abundant proteins (LEA) and stress-responsive transcription factors [6,27]. In this study, the overexpression of *SlAREB1* in tomato plants resulted in improved tolerance to in vitro osmatic stress induced by high levels of sucrose in tissue culture media when compared with negative control transgenic lines (Table 1; Figure 1). At 200 mm sucrose level, transgenic plants overexpressing *SlAREB1* showed high mean values for different plant growth parameters, including plant height, indicating the ability of transgenic plants to overcome the growth retardation effect induced by sucrose. Normally, the elevated levels of sucrose induce an osmotic stress that inhibits growth of in vitro cultured plants and extend the subculturing interval due to their ability to reduce cell osmotic potential, restriction of water availability and reduction of cell expansion and division [28]. However, this was not the case with transgenic tomato plants overexpressing *SlAREB1*, indicating that slow growth preservation by using sucrose for prolonged periods might be inappropriate for drought tolerant transgenic plants.

On the contrary, the utilization of ABA for slow growth preservation of in vitro cultured transgenic plants proved to be more effective when compared with sucrose treatment. For instance, severe growth retardation phenotypes were observed when microshoots of all tested transgenic lines were cultured on MS media supplemented with 8 or 12 µm ABA. This is consistent with previous studies where microshoots cultured on media containing high ABA levels resulted in growth inhibition and enabled long-term in vitro preservation [28,29,30]. On the other hand, transgenic line SlAREB1#3, producing high expression levels of *SlAREB1*, showed higher sensitivity to ABA at 4 µm concentrations after 12 weeks of culture as reflected in growth retardation phenotypes when compared with negative control (Table 3). This is somehow expected as the overexpression of *SlAREB1* homolog in different plant species resulted in enhanced sensitivity to ABA. For instance, the overexpression of *OsbZIP23* in transgenic rice plants resulted in higher sensitivity to ABA when compared with wild type plants [31].

Cryopreservation of tomato plant was reported previously by using different explant types including seeds [22,23], pollens [32], meristems [33] and shoot tips [17,18]. Using encapsulation-dehydration, shoot tips of different tomato genotypes were successfully cryopreserved with the highest regrowth rate obtained with cultivar Pontica precultured on 0.5 M sucrose for 1 day and dehydrated for 3 h [17]. Consistent with these results, the survival and regrowth of non-cryopreserved shoot tips of negative control transgenic lines decrease with increasing sucrose concentrations, which was more pronounced with longer dehydration periods (Table 4). This was attributed to higher osmotic stress conditions imposed by high sucrose levels, duration of pre-treatment and dehydration duration that would result in cell death and reduced regrowth of cryopreserved shoot tips [17]. On the contrary, non-cryopreserved transgenic plants overexpressing *SlAREB1* showed higher survival and regrowth rates when compared with negative control shoot tips and this could be attributed to their ability to tolerate osmotic shocks resulting from high sucrose levels and dehydration treatments. This is somehow expected, knowing that transgenic plants overexpressing *SlAREB1* showed improved growth under high sucrose concentrations (this study) and their ability to improve osmotic stress tolerance in transgenic tomato plants [6]. Such an approach seems promising, knowing that transgenic chrysanthemum plants with increased trehalose 6-phoshapte accumulation were also found to have improved tolerance to cryopreservation treatments [26].

Cryopreservation using aluminium V-cryoplates was developed for clonally propagated crops to ensure very high cooling and warming rates of treated explants [34]. The V-cryoplates procedure is known to produce high survival of cryopreserved plant tissues when compared with other methods [35]. In general, several studies reported that the preconditioning of shoot tips for 1 day in V-cryoplate experiments will produce very high recovery rates [34,35]. This is attributed mainly to the ability of in vitro tissues to adhere on the aluminium plates, resulting in a very rapid cooling after immersion in liquid nitrogen and very high warming rates in the unloading solution [20,36]. As a result, very high survival and regrowth rates with various plant species have been obtained after cryopreservation by using the cryoplates method [19]. In this study, transgenic tomato explants cryopreserved by using the V-cryoplates method were able to survive the liquid nitrogen treatments when compared with encapsulation-dehydration treated shoot tips. No major differences in survival percentages were observed in cryopreserved (with liquid nitrogen) negative control shoot tips subjected to different cryoplates treatments. On the contrary, transgenic plants overexpressing *SlAREB1* showed higher survival rates when subjected to a longer pretreatment incubation period for 3 days with or without cold acclimatization (Figure 6). This indicates the role of *SlAREB1* in improving tolerance against cryopreservation stressful treatments and its ability to improve tolerance to associated osmotic stress conditions.

The results of the present study revealed that after cryopreservation (liquid nitrogen exposure) by using encapsulation-dehydration, neither survival nor regrowth were obtained in all tested tomato lines. On the other hand, no regrowth was obtained after cryopreservation by using V-cryoplate methods; although regrowth was achieved successfully after cryopreservation in different tomato genotypes subjected to encapsulated-dehydration and droplet vitrification techniques, the regeneration potential of treated shoot tips was found to be genotype-dependent [17]. Furthermore, the decline in the survival and recovery rates of cryopreserved tomato in this study might be attributed to the formation of extra-cellular and intra-cellular ice crystals as a result of high moisture content of the cryopreserved tissues [37]. High moisture content was found to cause death of the encapsulated African violet (*Saintpaulia ionantha*) shoot tips that were pretreated with 0.1 M sucrose for 2 days before (8 h) dehydration [38]. Consistent with the results of this study, Sharaf et al. (2012) [39] reported a complete survival when encapsulated non-cryopreserved *Artemisia herba-alba* shoot tips were pretreated in 0.5 M sucrose for three days but low regrowth rates (27%) were observed. In conclusion, the low survival and recovery percentages after cryopreservation by using encapsulation-dehydration and V-cryoplates could be attributed to partial damage of the shoot tips due to osmotic shock after rehydration and ice crystallization [40], or might be due to unfavorable growth conditions after liquid nitrogen treatment [38].

The use of seed cryopreservation proved to be a convenient method over other tested methods in many plant species. For instance, the germination of cryopreserved *Encholirium pedicellatum* seeds was higher than control treated seeds [41]. In this study, higher survival rates and germination percentages were observed after seed cryopreservation when compared with encapsulation-dehydration and V-cryoplates. On the other hand, a clear genotypic effect was observed, with negative control and SlAREB1#2 being the fastest in germination before and after cryopreservation when compared with SlAREB1#3, a line producing higher expression levels of *SlAREB1*. This is somehow expected, knowing that *SlAREB1* homologs were found to play a major role in controlling seed development and dormancy in different plant species [42,43].

## 4. Materials and Methods

### 4.1. Cloning of SlAREB1 Gene

Based on DNA sequence information (GenBank accession number: AY530758), two gene-specific primer pairs were designed and used to clone *SlAREB1* from tomato plants as described in Orellana et al. (2010) [6]. To isolate the full length cDNA of the *SlAREB1*, seeds of tomato cv. Moneymaker were soaked in water for 2 days at 25 °C and then washed with sterilized water before sowing into small pots (10 cm diameter × 10 cm depth) filled with peat moss. After germination, tomato seedlings were grown for two weeks under controlled conditions (continuous 25 °C temperature, photoperiod of 16 h light/8 h dark with 80 µmol·m^−2^·s^−1^ photon flux density) and irrigated daily with fixed volume of Hoagland solution. Total RNA was isolated from leaf tissue of the two-week old seedlings using SV Total RNA Isolation System Kit (Promega Corporation, Madison, WI, USA) following the manufacturer′s instructions. The isolated RNA was used to synthesize a first strand cDNA library using the SuperScript^®^ First-Strand Synthesis System (Invitrogen, Carlsbad, CA, USA) and oligo T(_18_) primer following the manufacturer's instructions. The full-length cDNAs of the *SlAREB1* members were amplified using PCR in a 25 µL reaction mixture containing 5 µL of cDNA as a template, 2.5 µL of dNTPs (100 µm), 5 µL of 5× PCR buffer, 0.5 µm of each primer and 0.25 µL of 5 U/µL GoTaq DNA polymerase (Promega Corporation, Madison, WI, USA). The PCR conditions were 94 °C for 5 min, followed by 40 cycles of 94 °C for 30 s, 55 °C for 1 min, and 72 °C for 1 min, and a final 10 min extension at 72 °C. The amplified PCR products were separated in a 1% agarose gel and stained with ethidium bromide. Positive PCR products were extracted from agarose gels using Wizard^®^ SV Gel and PCR Clean-Up System (Promega Corporation, Madison, WI, USA) and cloned into pGEM^®^-T Easy Vector System (Promega Corporation, Madison, WI, USA) following manufacturer's instructions. Positive recombinant plasmids that contained the full-length cDNAs were fully sequenced using an ABI 3730XL machine by Macrogen (Seoul, Korea).

### 4.2. Plant Material

Transgenic tomato lines, derived from the Moneymaker tomato cultivar, transformed with either a construct for the overexpression of the drought-responsive transcription factor *SlAREB1* gene or with an empty plasmid (negative control) were used in this study. For transgenic plant generation, the full length cDNA of *SlAREB1* was introduced into the binary plasmid pCABIMA1302 by replacing the *GFP* gene with the *SlAREB1*gene at the *Nco*I and *Bst*EII sites (pCAMBIA1302/SlAREB1). The introduced transgene was under the control of the *CaMV 35S* promoter to allow its overexpression in transgenic plants. An empty binary plasmid and pCAMBIA1302/*SlAREB1* were used for *Agrobacterium tumefaciens*-mediated transformation of the Moneymaker cultivar at the Ralph M. Parsons Foundation Plant Transformation Facility at UC Davis (http://ucdptf.ucdavis.edu/). Transgenic seeds from T1 plants were selected on MS medium [44] containing 50 mg/L hygromycin and lines showing 3:1 segregation for the antibiotic resistance were selected to get the T2 progeny plants. T2 plants seeds were further analyzed for transgene existence using PCR, segregation for antibiotic resistance plants and gene expression levels using RT-qPCR analysis. In addition, transgene copy number was estimated using RT-qPCR using *neomycin phosphoril-transferase II* (*nptII*) gene specific primers and the *SlActin* (*Solyc03g078400*) gene as internal control as described previously [45].

For *SlAREB1* expression analysis in transgenic plants, total RNA was isolated from leaf samples taken from positive transgenic plants using SV Total RNA Isolation System Kit (Promega, Madison, WI, USA) as described above. The isolated RNA from transgenic plants was used to synthesize a first strand cDNA library using the SuperScript^®^ First-Strand Synthesis System (Invitrogen, Carlsbad, CA, USA) and oligo T(_18_) primer as described above. Gene-specific primer pairs for *SlAREB1* (SlAREB1ExpFwd: 5′-GTTTAGGAGCCAGTGGGGTC-3′ and SlAREB1ExpRev: 5′-CTGCCTCCTTTCAACGACCT-3′) and *SlActin* (*Solyc03g078400*; SlActinFwd: 5′-CCTGTTCTCCTGACTGAGGC-3′ and SlActinRev 5’-TGCTCCTAGCGGTTTCAAGT-3′), used as the reference internal control for relative gene expression analysis was designed using Primer 3 Software. Quantitative real-time PCR (qRT-PCR) analysis was performed as described previously [45].

Two T2 homozygous lines (SlAREB1#2 and SlAREB1#3) having a single insertion event (reflected in *nptII* to *SlActin* ratios close to 1 as calculated in [46]) and showing variable levels of transgene expression as revealed by quantitative RT-PCR and single negative control plant (harboring an empty T-DNA) were selected and used in this study.

### 4.3. In Vitro Establishment of Transgenic Lines

Seeds of two transgenic tomato lines overexpressing (SlAREB1#2 and SlAREB1#3) and the empty plasmid (negative control) were surface sterilized by washing thoroughly under running tap water for 20 min with a few drops of mild detergent (Tween^®^-20). Then, the seeds were surface sterilized by soaking in an antiseptic solution of 3% sodium hypochlorite for 5 min (under the laminar-air flow cabinet), and then rinsed with autoclaved distilled water three times (5 min each). Seeds were then transferred into 70% (*v*/*v*) ethanol solution for 30 s and rinsed with sterile distilled water 3 times (5 min each). After that, sterile seeds of tested lines were transferred to full strength MS media supplemented with 100 mm sucrose, under normal growth conditions in controlled chambers with a daily photoperiod regime of 16 h light (photosynthetic photon flux density = 40–45 µmol·m^−2^·s^−1^) and 8 h dark at 24 ± 1 °C until full germination. Germinated plantlets were transferred to MS free hormone media for further growth and development.

### 4.4. Slow Growth Preservation

For slow-growth conservation experiments, shoot tips (1.0 cm long) were excised from one month-old tomato in vitro plantlet and then transferred to MS solid media (25 mL dispensed into 150 mm × 25 mm test tubes) supplemented with different concentrations of sucrose (0, 100, 200, 300 mm) or with different concentrations of ABA (0, 4, 8, or 12 µm). Data were recorded on plantlets from each treatment twice (after 6 weeks and after 12 weeks) during 12 weeks of culture for plant height, number of roots and number of leaves. At the end of the 12-week culturing period, the microshoots were transferred to fresh solid MS medium supplemented with 0.1 M sucrose and the regrowth percentages were recorded after 4 weeks.

### 4.5. Cryopreservation

Shoot tips (~0.3 cm in length) from each transgenic line and negative control were dissected and precultured aseptically into a hormone free MS solid medium supplemented with 0.3 M sucrose for 3 days under dark conditions. The cryopreservation of the shoot tips was performed using encapsulation dehydration and V-cryoplates as described previously [34,47].

For encapsulation dehydration, two modified liquid MS media were used: the first medium lacks CaCl_2_ and was supplemented with 0.3 M sucrose and 3% (*w*/*v* %) sodium alginate (Bio world (Dublin, OH, USA)), alginic acid sodium salt (C_6_H_7_O_6_Na)_n_), which was used for shoot tips coating. The second liquid MS media contained CaCl_2_ and was supplemented with 0.3 M sucrose and 100 mm CaCl_2_·2H_2_O, which was used for shoot tips polymerization. The pretreated shoot tips were initially soaked in the alginate containing MS media under aseptic conditions. After that, each shoot tip was pulled up by using a micropipette along with 125 µL of the alginate media and dipped directly into the CaCl_2_ containing media for 30 min with continuous stirring, to allow complete polymerization of shoot tips to form beads. Some produced beads were left without shoot tips for moisture content determination. The encapsulated shoot tips were then transferred onto MS liquid media supplemented with different concentrations of sucrose (0.4 M or 0.8 M) and shaken at 500 rpm under dark conditions for either 1 or 3 days. After the end of beads incubation period, the encapsulated shoot tips were placed over filter paper in uncovered petri-dishes and dehydrated for 0, 3, or 6 h under aseptic conditions. Beads moisture content was measured by weighing the beads after each dehydration period (fresh weight); and after they were dried in oven (80–90 °C) for 18 h (dry weight). The moisture content (MC%) was determined by the following formula: MC% = [(Beads fresh weight—Beads dry weight)/Beads fresh weight] × 100. Thereafter, half of the dehydrated beads were transferred to 2 mL sterile cryovials and dipped into liquid nitrogen for at least 1 h at −196 °C, while the other half were transferred to 2 mL sterile cryovials but left at room temperature. The liquid nitrogen treated-cryovials were thawed in a water bath at 38 °C for 3 min.

For V-cryoplate experiments, shoot tips (~0.3 cm length) of each in vitro tomato transgenic line were dissected under sterilized conditions and precultured at 25 °C for 1 or 3 days on hormone free MS media supplemented with 0.3 M sucrose and incubated in the dark in growth room conditions as mentioned before. Another preculture treatment with prechilling at 4 °C for three days was also studied. After that, the precultured shoot tips were placed on aluminium V-cryoplates with 10 wells and embedded in alginate gel, then covered by CaCl_2_ for 15 min until full polymerization. Osmoprotection was performed by immersing the cryoplates for 20 min in loading solution (2 M glycerol + 1.0 M sucrose). For dehydration, the cryoplates were transferred and immersed in PVS2 vitrification solution (HF-MS + 30% glycerol + 15% ethylene glycol (EG) + 15% dimythelsulfoxide (DMSO) and 0.4 M sucrose) for 20 min at 25 °C. Then, the V-cryoplates were either kept at room temperature (non-cryopreserved) or transferred into uncapped 2 mL cryotubes and directly plunged into liquid nitrogen (cryopreserved). For cryopreservation treatment, the shoot tips were rewarmed by immersing the V-cryoplates in cryotubes containing 2 mL of unloading solution (1 M sucrose) at room temperature.

Viability of the shoot tips was assayed by using the 2, 3, 5-triphenyltetrazolium chloride salt solution (TTC) test as described previously [37]. For this purpose, encapsulated shoot tips (4 shoot tips/cryotube) were immersed with 2 mL of the TTC solution and kept in complete darkness for 16 h at 24 ± 1 °C. The shoot tips were then examined under binocular microscope (Leica microsystem, EZ4HD, Singapore) to obtain the survival percentage of each treatment. The following formula was used to measure the survival percentages of the treated shoot tips: survival percentage = (number of red shoot tips/total number of shoot tips) × 100%.

The cryopreserved and non-cryopreserved beads were transferred to a recovery MS media supplemented with 0.1 M sucrose and kept for 7 days under dark conditions. Afterwards, they were transferred to normal growth conditions and monitored for four weeks for any sign of regrowth (swelling, color change, leaf appearance, and shoot appearance). The regrowth percentage was calculated as the number of regrown encapsulated tips divided by the total number of encapsulated shoot tips × 100%.

For seed cryopreservation, a lot of up to 200 seeds of each transgenic line was dissected in special containers with silica gel to reduce the moisture content and to allow the seeds to reach an equilibrium state where initial fresh weight of seeds is equal to dry weight. After dissection, the seeds were used to study the effect of cryopreservation on seed germination by submerging screw-capped polypropylene cryovials (2 mL) into liquid nitrogen for at least 1 h. In addition, seeds were kept at room temperature without liquid nitrogen and used as non-cryopreserved checks. The treated seeds were then placed on filter paper in 9 cm petri dishes and kept at complete darkness at 25 °C. Seed germination was recorded for 4 intervals after 5, 7, 10, and 14 days.

### 4.6. Statistical Analysis

A Complete Randomized Design (CRD) was used in all experiments. For slow growth preservation, the treatments were replicated 7 times and the experiments were repeated twice. Meanwhile, 16 replications with 4 shoot tips per each replicate were used for the cryopreservation experiments that were divided as 8 replicates for re-growth examination, and 8 replicates for survival examination and the experiments were conducted once. For seed cryopreservation, 5 replicates with 10 seeds each were used and the experiment was repeated twice. All obtained results were statistically analyzed by using Statistical Analysis System (SAS) (ver. 9, SAS Inc., Cary, NC, USA) and ANOVA was obtained for each experiment. In case of significant interaction, the transgenic effect was tested within each period using the SLICE option. Mean separation was conducted by using Tukey′s HSD test at *p* < 0.05.

## 5. Conclusions

In conclusion, in vitro-grown tomato microshoots were successfully conserved under slow growth conditions using ABA that was able to reduce microshoot growth of transgenic lines and enabled regrowth after preservation. Using the encapsulation-dehydration technique, transgenic plants overexpressing *SlAREB1* showed better tolerance to severe osmotic stress conditions and higher survival and regrowth although no regrowth was observed after liquid nitrogen treatment. Similarly, transgenic plants overexpressing *SlAREB1* showed higher survival rates after liquid nitrogen treatment by using the V-cryoplate approaches, indicating the feasibility to use stress-related transcription factors to cryopreserve reluctant plant species. On the contrary, seed cryopreservation of transgenic plants overexpressing high levels of *SlAREB1* produced low germination percentages when compared with negative control, indicating a pleiotropic effect associated with activated ABA pathways.

## Figures and Tables

**Table 1:** Effects of sucrose concentration on plant height, roots number and leaves number of in vitro conserved microshoots of negative control, SlAREB1#2, SlAREB1#3 transgenic tomato plants after 6 and 12 weeks of storage.

Sucrose Concentration (mm)	Plant Height (cm)			Roots Number			Leaves Number		
	Control	SlAREB1#2	SlAREB1#3	Control	SlAREB1#2	SlAREB1#3	Control	SlAREB1#2	SlAREB1#3
	6 weeks								
0	1.00 a * ± 0.00 **	1.00 a ± 0.00	1.21 a ± 0.10	0.00 a ± 0.00	0.00 a ± 0.00	0.00 a ± 0.00	1.00 a ± 0.00	1.00 a ± 0.00	1.14 a ± 0.67
100	5.32 a ± 0.59	5.85 a ± 0.42	5.99 a ± 0.66	7.14 a ± 1.12	7.66 a ± 1.55	7.89 a ± 2.49	7.86 a ± 1.28	7.29 a ± 1.67	6.43 a ± 1.37
200	1.71 b ± 0.24	6.13 a ± 1.18	6.47 a ± 0.50	0.00 b ± 0.00	10.71 a ± 2.31	9.57 a ± 1.86	3.57 b ± 1.03	6.29 a ± 1.23	5.71 a ± 1.23
300	1.14 b ± 0.13	1.97 b ± 0.36	3.84 a ± 0.92	0.00 b ± 0.00	0.57 b ± 0.56	3.43 a ± 0.83	2.00 b ± 0.61	1.57 b ± 0.83	6.00 a ± 1.22
	12 weeks								
0	1.00 a ± 0.00	1.00 a ± 0.00	1.34 a ± 0.11	0.00 a ± 0.00	0.00 a ± 0.00	0.00 a ± 0.00	1.00 a ± 0.00	1.00 a ± 0.00	1.43 a ± 0.77
100	8.47 a ± 1.10	8.55 a ± 0.68	9.10 a ± 1.10	10.14 a ± 2.39	9.23 a ± 1.29	10.86 a ± 1.32	12.29 a ± 2.71	11.46 a ± 2.77	10.43 a ± 1.58
200	2.54 b ± 0.21	7.27 a ± 1.29	8.77 a ± 0.72	0.14 b ± 0.04	13.57 a ± 3.33	13.43 a ± 3.03	5.57 b ± 1.59	10.86 a ± 2.79	9.43 a ± 1.24
300	1.24 b ± 0.12	2.22 b ± 0.44	5.27 a ± 0.76	0.00 b ± 0.00	0.86 b ± 0.73	7.43 a ± 2.10	2.57 b ± 0.56	2.43 b ± 0.56	8.57 a ± 2.19

* Based on Tukey’s HSD test, different letter indicates significant difference among transgenic lines (*p* < 0.05) for each combination of sucrose concentration and number of weeks. ** Values are the mean ± SD.

**Table 2:** Regrowth percentages of in vitro conserved microshoots of negative control, SlAREB1#2, SlAREB1#3 transgenic tomato plants after 12 weeks from culturing on media with different levels of sucrose and abscisic acid (ABA).

Transgenic Lines	Sucrose Concentration (mm)				ABA Concentration (µm)			
	0.00	100	200	300	0	4	8	12
Control	0.00 a * ± 0.00 **	78.6 a ± 10.97	38.5 b ± 13.00	21.4 b ± 10.97	71.4 a ± 12.07	71.4 a ± 12.07	81.8 a ± 10.31	83.0 a ± 9.96
SlAREB1#2	0.00 a ± 0.00	81.8 a ± 10.31	71.4 a ± 12.07	38.5 b ± 13.00	76.9 a ± 11.26	78.6 a ± 10.97	84.6 a ± 9.64	83.0 a ± 9.96
SlAREB1#3	0.00 a ± 0.00	85.7 a ± 9.35	78.6 a ± 10.97	92.3 a ± 7.12	85.7 a ± 9.35	69.2 a ± 12.97	78.6 a ± 10.97	78.56 a ± 10.97

* Based on Tukey’s HSD test, different letter indicates significant difference among transgenic lines (*p* < 0.05) for each concentration. ** Values are the mean ± SD.

**Table 3:** Effects of ABA concentration on plant height, roots number and leaves number of in vitro conserved microshoots of negative control, SlAREB1#2, SlAREB1#3 transgenic tomato plants after 6 and 12 weeks of storage.

ABA Concentration (µm)	Plant Height (cm)			Roots Number			Leaves Number		
	Control	SlAREB1#2	SlAREB1#3	Control	SlAREB1#2	SlAREB1#3	Control	SlAREB1#2	SlAREB1#3
	6 weeks								
0	5.04 a * ± 0.55 **	5.14 a ± 1.31	5.01 a ± 0.70	5.86 a ± 1.42	6.57 a ± 2.01	5.57 a ± 1.03	6.14 a ± 1.42	5.71 a ± 1.58	5.29 a ± 1.00
4	2.91 a ± 0.66	2.46 a ± 0.37	2.11 a ± 0.31	5.43 a ± 1.81	4.57 a ± 1.20	1.86 b ± 1.55	5.14 a ± 1.42	5.29 a ± 1.80	2.86 b ± 1.13
8	1.41 a ± 0.11	1.43 a ± 0.15	1.53 a ± 0.25	0.00 a ± 0.00	0.14 a ± 0.40	0.00 a ± 0.00	2.71 a ± 0.52	3.14 a ± 0.95	3.00 a ± 0.86
12	1.46 a ± 0.20	1.51 a ± 0.13	1.43 a ± 0.18	0.00 a ± 0.00	0.29 a ± 0.80	0.00 a ± 0.00	2.57 a ± 0.83	3.29 a ± 0.80	3.14 a ± 0.40
	12 weeks								
0	7.19 a ± 1.00	7.33 a ± 1.75	6.93 a ± 0.56	9.86 a ± 2.69	8.71 a,b ± 1.18	7.57 b ± 2.10	13.00 a ± 3.56	11.71 a ± 2.91	11.00 a ± 1.83
4	6.90 a ± 1.19	7.14 a ± 1.61	2.26 b ± 0.32	8.43 a ± 2.79	6.86 a ± 0.95	1.86 b ± 1.55	9.71 a ± 2.42	8.29 a ± 1.58	3.86 b ± 1.42
8	1.53 a ± 0.15	1.94 a ± 0.29	1.60 a ± 0.15	0.14 a ± 0.40	0.57 a ± 0.83	0.14 a ± 0.40	5.43 a ± 0.83	5.14 a ± 0.95	4.43 a ± 0.83
12	1.73 a ± 0.27	1.73 a ± 0.26	1.61 a ± 0.20	0.00 a ± 0.00	0.29 a ± 0.52	0.00 a ± 0.00	5.29 a ± 0.80	4.14 a ± 0.73	3.71 a ± 1.00

* Based on Tukey’s HSD test, different letter indicates significant difference among transgenic lines (*p* < 0.05) for each combination of ABA concentration and number of weeks. ** Values are the mean ± SD.

**Table 4:** Germination percentages of non-cryopreserved and cryopreserved seeds of negative control, SlAREB1#2, SlAREB1#3 transgenic tomato plants.

Transgenic Lines	Non-Cryopreserved				Cryopreserved			
	Number of Days				Number of Days			
	5	7	10	14	5	7	10	14
Control	60 a * ± 7.07 **	80 a ± 7.07	84 a ± 5.47	100 a ± 0.00	60 a ± 7.07	78 a ± 7.07	86 a ± 5.47	100 a ± 0.00
SlAREB1#2	50 a ± 12.24	72 a ± 4.47	78 a ± 5.47	94 a ± 5.47	60 a ± 12.24	72 a ± 4.47	76 a ± 5.47	96 a ± 8.49
SlAREB1#3	25 b ± 7.07	44 b ± 5.47	50 b ± 10.00	54 b ± 8.49	30 b ± 7.07	42 b ± 5.47	50 b ± 10.00	74 b ± 5.47

* Based on Tukey’s HSD test, different letter indicates significant difference among transgenic lines (*p* < 0.05) for each concentration. ** Values are the mean ± SD.

## Supplementary Materials

Supplementary materials can be found at www.mdpi.com/1422-0067/18/7/1477/s1.

## Abbreviations

ABA	Abscisic acid
AREB	*ABA responsive element* binding
LN	Liquid nitrogen
MS media	Murashige and Skoog Medium
PVS2	Plant Vitrification solution 2
PCR	Polymerase chain reaction
RT-PCR	Real time-Polymerase chain reaction
SPSS	Statistical Package for Social Sciences
TTC	2,3,5-Triphenyltetrazolium chloride salt solution
V-cryoplates	Vitrification-cryoplates
