# Supplementary material for: In Vitro Preservation of Transgenic Tomato (Solanum lycopersicum L.) Plants Overexpressing the Stress-Related SlAREB1 Transcription Factor
Source: Int J Mol Sci. 2017 Jul 21;18(7):1477. doi: 10.3390/ijms18071477 (PMC5535968; doi:10.3390/ijms18071477)
Supplement: Supplementary file 1 [file ijms-18-01477-s001.pptx]

## Slide 1
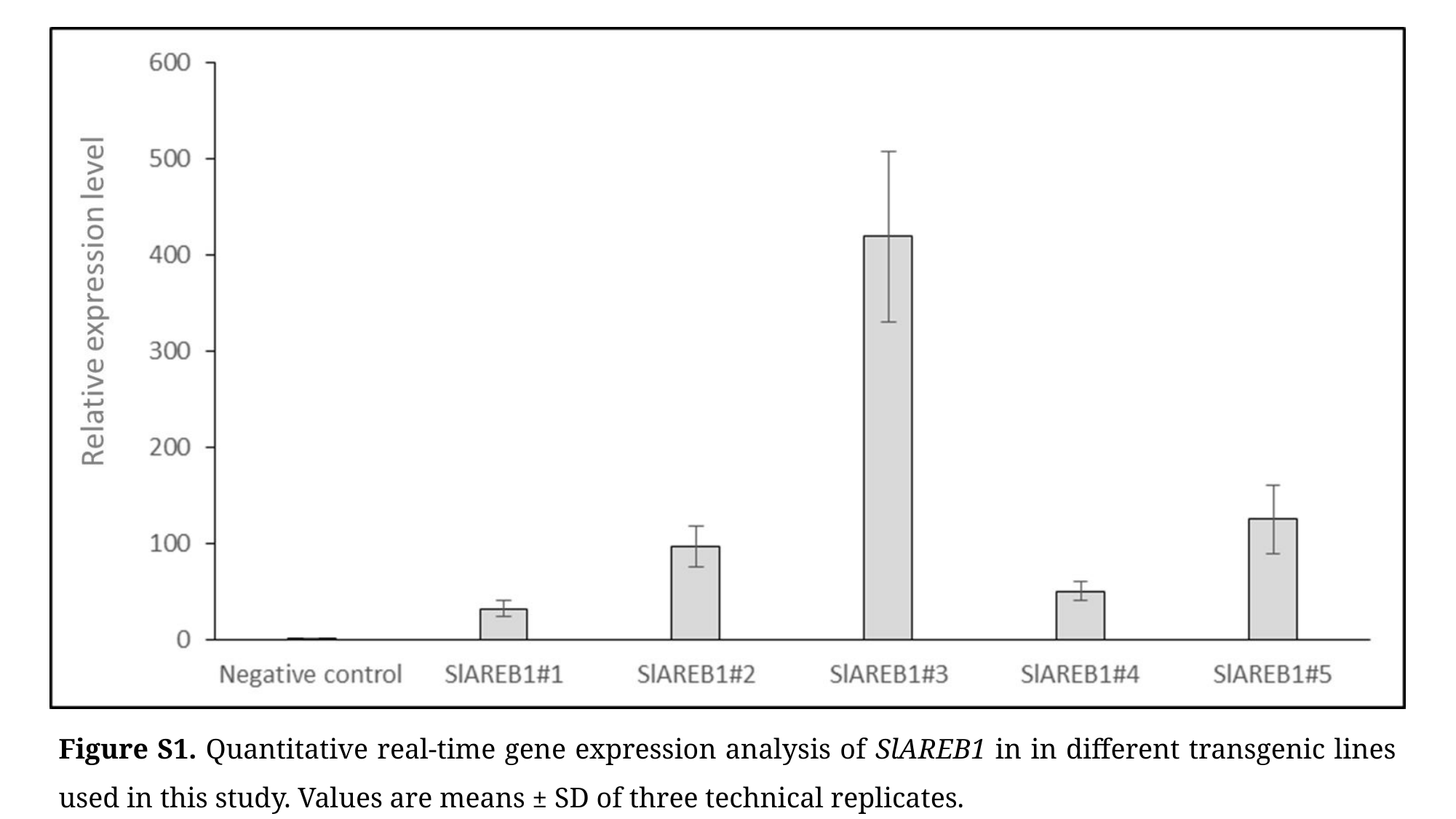

Figure S1. Quantitative real-time gene expression analysis of SlAREB1 in in different transgenic lines used in this study. Values are means ± SD of three technical replicates.

## Slide 2
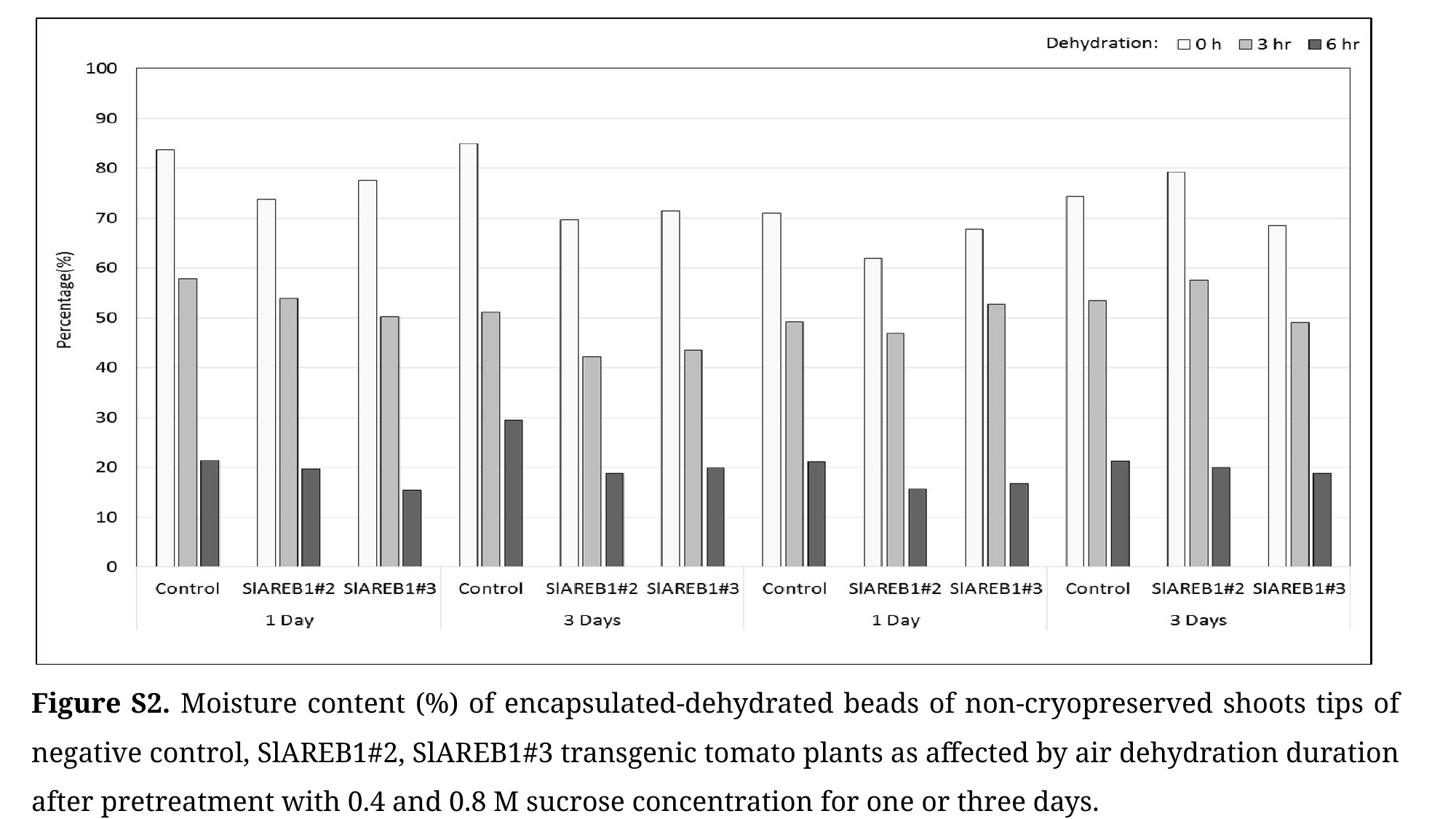

Figure S2. Moisture content (%) of encapsulated-dehydrated beads of non-cryopreserved shoots tips of negative control, SlAREB1#2, SlAREB1#3 transgenic tomato plants as affected by air dehydration duration after pretreatment with 0.4 and 0.8 M sucrose concentration for one or three days.
